# Using resource modelling to inform decision making and service planning: the case of colorectal cancer screening in Ireland

**DOI:** 10.1186/1472-6963-13-105

**Published:** 2013-03-19

**Authors:** Linda Sharp, Lesley Tilson, Sophie Whyte, Alan O Ceilleachair, Cathal Walsh, Cara Usher, Paul Tappenden, James Chilcott, Anthony Staines, Michael Barry, Harry Comber

**Affiliations:** 1 National Cancer Registry Ireland, Cork Airport Business Park, Kinsale Road, Cork, Ireland; 2 National Centre for Pharmacoeconomics, St James’s Hospital, Dublin, Ireland; 3 School for Health and Related Research, University of Sheffield, Sheffield, UK; 4 Department of Statistics, Trinity College Dublin, Dublin, Ireland; 5 School of Nursing, Dublin City University, Dublin, Ireland

**Keywords:** Colorectal cancer, Adenomatous polyps, Mass screening, Resource utilization, Markov chains

## Abstract

**Background:**

Organised colorectal cancer screening is likely to be cost-effective, but cost-effectiveness results alone may not help policy makers to make decisions about programme feasibility or service providers to plan programme delivery. For these purposes, estimates of the impact on the health services of actually introducing screening in the target population would be helpful. However, these types of analyses are rarely reported. As an illustration of such an approach, we estimated annual health service resource requirements and health outcomes over the first decade of a population-based colorectal cancer screening programme in Ireland.

**Methods:**

A Markov state-transition model of colorectal neoplasia natural history was used. Three core screening scenarios were considered: (a) flexible sigmoidoscopy (FSIG) once at age 60, (b) biennial guaiac-based faecal occult blood tests (gFOBT) at 55–74 years, and (c) biennial faecal immunochemical tests (FIT) at 55–74 years. Three alternative FIT roll-out scenarios were also investigated relating to age-restricted screening (55–64 years) and staggered age-based roll-out across the 55–74 age group. Parameter estimates were derived from literature review, existing screening programmes, and expert opinion. Results were expressed in relation to the 2008 population (4.4 million people, of whom 700,800 were aged 55–74).

**Results:**

FIT-based screening would deliver the greatest health benefits, averting 164 colorectal cancer cases and 272 deaths in year 10 of the programme. Capacity would be required for 11,095-14,820 diagnostic and surveillance colonoscopies annually, compared to 381–1,053 with FSIG-based, and 967–1,300 with gFOBT-based, screening. With FIT, in year 10, these colonoscopies would result in 62 hospital admissions for abdominal bleeding, 27 bowel perforations and one death. Resource requirements for pathology, diagnostic radiology, radiotherapy and colorectal resection were highest for FIT. Estimates depended on screening uptake. Alternative FIT roll-out scenarios had lower resource requirements.

**Conclusions:**

While FIT-based screening would quite quickly generate attractive health outcomes, it has heavy resource requirements. These could impact on the feasibility of a programme based on this screening modality. Staggered age-based roll-out would allow time to increase endoscopy capacity to meet programme requirements. Resource modelling of this type complements conventional cost-effectiveness analyses and can help inform policy making and service planning.

## Background

Various international organisations have recommended screening for colorectal cancer [1,2]. While screening programmes are in place, or being piloted, in several European countries [3], many other countries have not yet implemented screening. Several screening tests are available. These include endoscopy-based tests (such as colonoscopy and flexible sigmoidoscopy (FSIG)), radiology-based tests (such as barium enema and CT colonography) and faecal tests (such as the guaiac-based faecal occult blood (gFOBT) and faecal immunochemical test (FIT)). Each test has its own strengths and limitations [4]. In addition, while most cost-effectiveness analyses suggest that organised screening - by most of the available tests - is likely to be considered cost-effective, none of the tests appears optimal across all settings [5]. This creates considerable uncertainty for those tasked with making policy and providing services.

Cost-effectiveness is only one of the issues which needs to be considered when taking decisions on screening implementation. Long-established criteria from the World Health Organisation (WHO) recommend also considering the safety of the screening test and associated diagnostic investigations, the acceptability of the test, and availability of diagnosis and treatment facilities [6]. The challenge for policy-makers is, therefore, how to take all of these issues into account in weighing screening options against one another to determine the “best” strategy in a given setting. The challenge for health service planners is slightly different: it concerns how to deliver the screening tests and associated health services required for diagnosis, treatment and follow-up. Both of these challenges are further complicated by demographic changes in western populations, which mean that the population in the screening age-range is projected to rise. Moreover, screening tests which are repeated regularly (e.g. faecal tests) impact on the eligible population, since people diagnosed with cancer or adenoma(s) are no longer eligible to be invited for screening. Thus consideration needs to be given to capacity requirements immediately upon programme implementation and over the coming five, ten or twenty years. Cost-effectiveness analyses, with results expressed in costs per quality adjusted life year gained (QALY) or life years saved (LYS), may not help health service managers address such practical issues [7]. Other types of analyses, which express the real, immediate, impact on the health services of actually introducing a screening programme (such as budget impact [8] or other similar analyses) may be of more use. However, these other types of analyses are rarely reported.

We conducted a cost-effectiveness analysis of three strategies for colorectal cancer screening in Ireland: biennial gFOBT at ages 55–74, biennial FIT at ages 55–74 and once-only FSIG at age 60. All three options were considered to be highly cost-effective compared to the *status quo* of no screening. The cost per QALY gained was €589 for FSIG, €1,696 for FIT and €4,428 for gFOBT [9]. FIT-based screening was expected to result in the greatest gain in QALYs and largest reductions in cancer incidence and mortality once fully implemented (i.e. the population cohort had completed 10 screening rounds). However, it was associated with major resource requirements over the lifetime of the cohort and higher rates of screening-related harms, including bowel perforations and deaths, than the other options. These results presented a significant challenge for decision-makers and service providers, raising questions about the feasibility, practicality and acceptability of a programme based on screening individuals aged 55–74 by biennial FIT. For example, there were questions about the actual resources required for diagnosis and treatment (for example, the numbers of colonoscopies which would need to be conducted), whether the service could deliver these, and the impact of higher than estimated uptake. Safety was also a concern, with questions about the actual number of people expected to suffer adverse effects of screening each year, and whether this was likely to be within acceptable limits. These questions stimulated queries about whether taking a different approach to screening delivery would make a programme based on FIT more feasible: for example, initially offering screening to a restricted age-group with gradual roll-out to incorporate the full age-range (as has been done in England in relation to the roll-out of the gFOBT-based screening programme). These types of questions cannot be answered by a conventional cost-effectiveness analysis.

In this paper we describe how we used a Markov state-transition model to estimate health service resource requirements and health outcomes, relative to the actual population of Ireland, over the first decade of a population-based colorectal cancer screening programme. These analyses were done with a view to aiding decision-making and service planning, and intended to complement the cost-effectiveness analyses.

## Methods

### Setting and screening scenarios

The country of Ireland is a republic comprising the largest part of the island of Ireland (the remainder of the island is Northern Ireland). The country has a population of 4.4 million, 700,800 of whom are aged 55–74. The healthcare system involves a mixture of public and private provision. Cancer screening programmes are provided free at the point of delivery, so the costs of provision fall on the healthcare payer, in this instance the Health Service Executive (HSE).

This analysis was part of a health technology assessment (HTA) of population-based colorectal cancer screening in Ireland. The commissioners, the Health Information & Quality Authority, established an Expert Group to scope the HTA, and recommend which screening modalities and age-groups should be evaluated [10]. These decisions were informed by: the available scientific evidence at the time, age-specific colorectal cancer incidence and mortality in Ireland, and considerations of feasibility and likely acceptability. The three base-case scenarios recommended for evaluation were: (1) biennial gFOBT, with reflex FIT (i.e. those with a weak positive gFOBT would be invited to complete a FIT), in individuals aged 55–74 years; (2) biennial FIT in individuals aged 55–74 years; and (3) once-only FSIG at age 60. Investigation of positive screening tests was assumed to be by colonoscopy or, in those unfit for colonoscopy, by CT colonography. Follow-up of those who had adenomas detected and removed was assumed to follow existing guidelines [11]. For gFOBT and FIT, we assumed that approximately half the population aged 55 or 56 would be invited to participate in year one of the programme and the remainder in year two, and similarly for those aged 57 or 58, and so on. Thus biennial screening in the 55–74 age group involved a maximum of ten screening rounds.

Since FIT-based screening was considered the optimal option in terms of cost-effectiveness, health service requirements associated with alternative scenarios for implementing FIT-screening were also considered. The two main alternative implementation approaches involve either restrictions by area or by age. The former is considered unacceptable by the National Cancer Screening Service (NCSS) in Ireland, so only age-based implementation strategies were considered. The first variant involved restricting screening to the 55–64 age-group (i.e. a maximum of five screening rounds). The second and third variants involved staggered roll-out of biennial screening in the 55–74 age-group. “Medium implementation” involved inviting those aged 55 and 65 in year one; those aged 55, 57, 65 and 67 in year two; those aged 55, 57, 59, 65, 67 and 69 in year three; and so on until the full age range would be included in year five. “Slow implementation” involved inviting those aged 55 in year one; those aged 55 and 57 in year two, those aged 55, 57 and 59 in year three; and so on until year ten when the full age-range would be included. These scenarios were not intended to represent the only or preferred options, but rather were chosen to illustrate the health service impact of different possible approaches to implementation.

### Estimating resource use and health outcomes

“Conventional” analysis of a Markov model - for the purposes of cost-effectiveness analysis - follows a single notional cohort, usually comprising 100,000 individuals, over their lifetime accumulating costs and benefits as the cohort ages. The approach adopted for this analysis was similar to that taken in a budget impact analysis [8]. We considered a short time horizon - the first 10 years following screening implementation. We estimated requirements separately for a range of resources (from screening tests to colorectal resections). We expressed our results in relation to the actual target population in Ireland. To do this, we ran our economic model, described below, using a cross-sectional whole population approach. We established 70 different age-cohorts, corresponding to the 2008 Irish population; cohort one comprised individuals aged 30, cohort two comprised individuals aged 31, and so on. We followed these 70 age-cohorts through the model for 10 years. For each cohort, the screening-related resources required (e.g. screening tests, diagnostic tests, etc.) and health outcomes experienced (e.g. numbers diagnosed with adenomas, had bowel perforations) were estimated, then summed across the whole population (i.e. all 70 cohorts) for each year following screening implementation. The predictions incorporated changes in the population age distribution over time and lower cancer prevalence in later screening rounds.

Resources associated with screening were assumed to be provided by the HSE and, within that, the NCSS. The health service resources estimated included those associated with: screening (gFOBT and FIT kits dispatched and returned; FSIG examinations); and diagnosis and treatment of screen-detected lesions (up to and including the point of surgery) and surveillance of individuals with screen-detected adenomas (colonoscopy, CT colonography, pathology, diagnostic radiology, neo-adjuvant radiotherapy (with or without chemotherapy) and colorectal resection). The health outcomes considered were the numbers of individuals who were diagnosed with screen-detected adenomas and cancers and the numbers who sustained complications (major abdominal bleeding requiring hospital admission, bowel perforation, death following perforation).

### Model

We used a Markov state-transition model which is described in detail elsewhere [9,12]. Briefly, the model included three interlinked components relating to: (a) the natural history of colorectal neoplasia; (b) screening scenarios, and subsequent adenoma surveillance; and (c) mortality. The natural history model simulated the experience of a cohort of individuals over their lifetime (from age 30 to 100) through a finite number of defined health states relating to the progression from having normal colorectal epithelium though the adenoma-carcinoma sequence to death (Additional file 1: Figure S1). The screening intervention model was superimposed upon the natural history model and the impact of the screening and diagnostic tests, and management of adenomas and cancers, modelled by re-distributing the cohort across health states in each Markov cycle. The mortality model allowed for deaths due to colorectal cancer, endoscopic bowel perforation, and other causes.

### Parameters and calibration

To populate the model, comprehensive literature reviews were undertaken. These were augmented with data and results from population-based screening programmes and pilot programmes. When relevant data was unavailable, values were based on expert clinical opinion. Table 1 shows the parameter values and the sources from which they were derived [13-49].

**Table 1:** Parameter estimates

***Model parameter***	***Base-case value***	***Lower and upper values in sensitivity analysis***	***References***
*Performance of screening tests*			
gFOBT sensitivity for adenomas	11%	10%, 12%^1^	[13-21]
gFOBT sensitivity for CRC	36%	31%, 42%^1^	
gFOBT specificity for adenomas and CRC	97%	96%, 98%	
FIT sensitivity for adenomas	21%	19%, 22%^1^	[16,22-32]
FIT sensitivity for CRC	71%	67%, 75%^1^	
FIT specificity for adenomas and CRC	95%	94%, 96%	
FSIG sensitivity for low-risk distal adenomas	65%	60%, 70%^1^	Expert opinion informed by [18,20,22]
FSIG sensitivity for intermediate/high-risk distal adenomas	74%	68%, 78%^1^	
FSIG sensitivity for distal CRC	90%	85%, 95%^1^	
FSIG specificity for distal adenomas and CRC	92%	90%, 95%	
*Uptake and compliance with screening, diagnosis and surveillance*			
gFOBT uptake	53%	32%, 70%	[34-37]
FIT uptake	53%	32%, 70%	
FSIG uptake	39%	24%, 67%	[38-40]
COL compliance (diagnostic test or adenoma surveillance)	86%	-	[34,35]
*Performance of diagnostic tests and related parameters*			
COL sensitivity for low-risk adenomas	77%	-	[41,42]
COL sensitivity for intermediate/high-risk adenomas	98%	-	
COL sensitivity for CRC	98%	-	
COL specificity for adenomas and CRC	97%	-	Expert opinion
CTC sensitivity for low-risk adenomas	53%	-	Expert opinion, informed by [43-46]
CTC sensitivity for intermediate/high-risk adenomas	85%	-	
CTC sensitivity for CRC	85%	-	
CTC specificity for adenomas and CRC	86%	-	
Average number of adenomas removed per person	1.9	-	[47]
% of those with intermediate/high-risk adenomas removed in whom the adenoma was high-risk	29%	-	[34]
*Harms of screening*			
FSIG probability of perforation (with or without polypectomy)	0.002%	-	[38]
FSIG probability of death following perforation	6.452%	-	[48]
Probability of (major) bleeding following FSIG	0.029%	-	[38]
COL probability of perforation (with polypectomy)	0.216%	-	[49]
COL probability of perforation (without polypectomy)	0.107%	-	
COL probability of death following perforation	5.195%	-	[48]
Probability of (major) bleeding following COL	0.379%	-	[38]

*COL* colonoscopy, *CRC* colorectal cancer, *CTC* CT colonography, *FIT* faecal immunochemical test, *gFOBT* guaiac faecal occult blood test, *FSIG* flexible sigmoidoscopy; low-risk adenoma(s), <10 mm; intermediate/high-risk adenoma(s), ≥10 mm. ^1^ parameters varied simultaneously in sensitivity analysis; - parameters not varied in sensitivity analyses.

Base-case estimates of screening uptake and colonoscopy compliance came from the UK pilot colorectal cancer screening programmes and the UK FSIG trial [34,35,38]. GFOBT and FIT uptake were assumed to be the same and not to change over screening rounds. Compliance with post-polypectomy surveillance was assumed to be the same as with diagnostic colonoscopy. GFOBT and FIT sensitivity and specificity values were derived from pooled analysis of information from diagnostic cohort studies of “screening populations” (i.e. the diagnosis had not been determined prior to recruitment and all participants underwent the index test and the reference “gold-standard” test) and, for gFOBT, which used Hemoccult® or Hemoccult II® [13-32]. While we did not set out to evaluate FIT at different cut-off values (because of lack of high-quality data at the time the model was parameterized) the key studies which informed the parameter estimates for FIT performance characteristics used a cut-off of 100 ng/mL. Three studies were combined to estimate FSIG sensitivity for intermediate/high-risk adenomas (adenomas ≥10 mm in size) [18,20,33]. Estimates of sensitivity for low-risk adenomas (<10 mm) and cancers, and specificity, were based on expert clinical opinion; these estimates were based on the assumption that sensitivity for low-risk adenomas would be lower than that for high-risk adenomas and sensitivity for cancer would be higher than that for high-risk adenomas. Colonoscopy performance characteristics were based on “miss rates” from studies of individuals who had undergone tandem colonoscopies [41,42]. CT colonography performance characteristics were based on expert opinion informed by reviews and large-scale studies [43-46]. It was assumed that, on average, 1.9 adenomas would be removed from each person undergoing polypectomy [47]. The probability of perforation following FSIG was derived from the UK FSIG Trial [38]. For colonoscopy, probabilities of perforation (with and without polypectomy) were estimated from a Swedish nationwide colonoscopy audit [49]. Estimates of the probability of death following perforation came from Gatto et al. [48]. Estimates of major bleeding following endoscopy procedures were from the UK FSIG Trial [38].

Several parameters, including natural history transition probabilities, could not be empirically observed and were obtained by calibration: This involved fitting the model to colorectal cancer incidence (by stage) and mortality data for Ireland. Further details of the approach and the transition probabilities are provided elsewhere [9,50]. The colorectal cancer incidence and mortality figures predicted by the model were very close to the observed values [9].

### Analysis

The primary analysis concerned absolute screening impact (i.e. annual numbers of each health service resource required, and each health outcome, under each screening scenario). One-way sensitivity analyses explored the impact of varying (a) screening uptake, (b) screening test sensitivity and (c) screening test specificity. Values for lower and higher uptake were based on experience in other countries [36,37,39,40]. For test sensitivity and specificity, the values used were the lower and upper 95% confidence intervals obtained from the pooled data used to derive the base-case values.

The secondary analysis assessed the relative impact of screening. For each core screening scenario, we estimated the additional resources needed for diagnosis and treatment, and the population-level health gains, compared to those required or generated under a policy of no screening.

## Results

### Primary (absolute) analysis of base-case scenarios

The model predicted that, in year one of a programme based on gFOBT or FIT, 357,812 individuals would be sent test kits, and 189,640 completed kits would be returned for laboratory processing (Table 2). With a FSIG-based programme, in year one, an estimated 18,617 individuals would undergo screening. Assuming uptake remained constant, between years one and 10, the number screened by FIT or gFOBT would increase by 16-17% and by FSIG by 11%, due to demographic changes.

**Table 2:** Estimated screening-related resource use and health outcomes by year and screening scenario: biennial gFOBT at 55–74 years, biennial FIT at 55–74 years, and FSIG once at 60 years

***Year of programme***			***Year 1***			***Year 10***		
***Screening scenario***			***gFOBT***	***FIT***	***FSIG***	***gFOBT***	***FIT***	***FSIG***
**Screening-related resource use**								
***Screening tests***	No. of kits sent out		357,812	357,812		420,151	417,464	
	No. of kits processed		189,640	189,640		222,637	220,999	
	No. of FSIG done^1^				18,617			20,625
***COL/CTC***	No. of diagnostic COL		967	11,095	381	1,103	12,414	423
	No. of diagnostic CTC		126	1,442	50	143	1,614	55
	No. of surveillance COL		0	0	0	297	2,406	620
	No. of surveillance CTC		0	0	0	39	313	81
***Pathology***	No. of adenomas and CRCs requiring pathology^2^		1,004	7,161	1,599	1,356	8,909	2,222
***CRC work-up and treatment***	No. receiving PET scan		31	85	6	34	69	8
	No. receiving MRI scan		111	307	23	121	247	28
	No. receiving CT scan(s)		309	853	64	336	687	78
	No. receiving TUS		16	43	3	17	35	4
	No. receiving pre-operative radiotherapy^3^		71	196	15	75	146	17
	No. undergoing colorectal resection^4^		281	779	59	307	635	71
**Screening-related health outcomes**								
***Harms***^***5***^	No. with major bleeding following endoscopy		4	48	7	6	62	10
	No. with perforation following endoscopy		2	21	1	2	27	2
	No. of deaths from perforation following endoscopy		0	1	0	0	1	0
***Screen-detected lesions***	No. with adenoma(s)^6^	Total	366	3,320	808	537	4,327	1,128
	*low-risk*		*229*	*2,081*	*544*	*333*	*2,770*	*740*
	*intermediate/high-risk*		*137*	*1,239*	*264*	*204*	*1,558*	*388*
	No. with CRC^7^	Total	309	853	64	336	687	78
	*stage I*		*111*	*308*	*23*	*130*	*303*	*31*
	*stage II*		*105*	*293*	*22*	*115*	*237*	*26*
	*stage III*		*69*	*192*	*14*	*68*	*115*	*16*
	*stage IV*		*24*	*60*	*4*	*23*	*31*	*5*

*COL* colonoscopy, *CRC* colorectal cancer, *CTC* CT colonography, *gFOBT* guaiac-based faecal occult blood test, *FIT* faecal immunochemical test, *FSIG* flexible sigmoidoscopy, *TUS* ultrasound; intermediate/high-risk = adenoma(s) ≥10 mm; low-risk = adenoma(s) <10 mm;

^1^ includes FSIG with and without polypectomy.

^2^ sum of number of adenomas and colorectal cancers requiring pathology, assuming average of 1.9 adenomas per person; includes screen-detected and surveillance-detected adenomas.

^3^ applies to rectal cancer only; includes radiotherapy given with or without chemotherapy.

^4^ sum of estimates of colon and rectal resections required.

^5^ includes complications from diagnostic and surveillance endoscopy, including FSIG where relevant.

^6^ includes individuals with screen-detected and surveillance-detected adenomas.

^7^ includes individuals with CRC detected at screening and at surveillance.

For FIT-based screening, in year one, resources would be required to perform 11,095 diagnostic colonoscopies and 1,441 CT colonographies, rising to 12,414 colonoscopies and 1,614 CT colonographies in year 10. The diagnostic resources required under gFOBT screening would be one-tenth of those required for FIT. With once-only FSIG, only 381–423 individuals would undergo diagnostic colonoscopy each year. Similar patterns were evident in the colonoscopy and CT colonography resources required for surveillance of those with intermediate/high-risk adenomas removed. As a consequence of the numbers of colonoscopies, more individuals would suffer adverse events with screening based on FIT, than with screening based on gFOBT or FSIG. With FIT, in year 10, an estimated 62 people would suffer major abdominal bleeding resulting in hospital admission, and there would be 27 bowel perforations and one associated death.

The yield of adenomas and cancers was predicted to be higher for FIT-based screening than the other two options, and therefore the resources required to manage these would be much greater. In year one, under FIT-based screening, 7,161 lesions (6,308 adenomas and 853 cancers) would require pathological analysis, compared to 1,599 (1,525 and 64) using FSIG, and 1,004 (695 and 309) using gFOBT. Resources would be required to conduct 774 resections of screen-detected cancers in year one with FIT screening, compared to 280 with gFOBT and 59 with FSIG. In year 10, these numbers would rise to 635, 307 and 71 for FIT, gFOBT and FSIG screening, respectively.

### Sensitivity analyses

**Figure 1:**
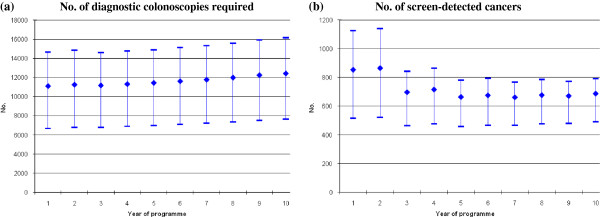
**Sensitivity analysis: estimated numbers of (a) diagnostic colonoscopies required and (b) screen-detected cancers, for years 1–10, with biennial FITat 55–74 years, as screening uptake varies around base-case value.** Numbers at base-case update shown as diamonds. Numbers under lower and higher update shown as dashes.

Numbers of diagnostic colonoscopies and screen-detected cancers estimated from the sensitivity analyses are shown in Additional file 2: Table S1 for all three screening scenarios, and in Figure 1 for biennial FIT at 55–74 years. Varying screening uptake had the greatest impact. For example, for FIT-based screening, compared to base-case uptake (53%), with low uptake (32%) the annual number of diagnostic colonoscopies required would be around 40% lower; with high uptake (70%) it would be almost one-third higher. Compared to base-case uptake, with low uptake 40% fewer cancers would be detected by screening in year one and 29% fewer in year 10; with high uptake, 32% more cancers would be detected in year one and 14% more in year 10. Varying specificity had a notable effect on colonoscopy requirements; for example, for FIT-based screening, in year one, high specificity (96%) increased the annual number of diagnostic colonoscopies by 13%, while low specificity (94%) reduced colonoscopy requirements by 13%. The impact of varying sensitivity was modest (1-6% variation around base-case values).

### Alternative FIT scenarios

Additional file 3: Table S2 shows the resource requirements and health outcomes for the three alternative implementation options for FIT-based screening. Resource requirements under the age-restricted option (FIT at 55–64 years) were approximately 60% of those required for screening the full age-range (e.g. 6,437-7,070 diagnostic colonoscopies annually vs 11,095-12,414 for the base-case). Only around half the number of cancers and adenomas would be detected under this scenario compared to screening the full age-range. Under the medium roll-out option for the full age-group, resource requirements would start low (2,441 diagnostic colonoscopies in year one vs 11,095 for the base-case scenario) rising to the same as those for the base-case by year 10. Numbers of adenomas and cancers detected followed a similar pattern. The slow roll-out option generated the lowest resource requirements in early years, reaching around 60% of the base-case requirement by year 10, but only 317 cancers would be detected in year 10 under this scenario compared to 687 under the base-case.

### Relative (secondary) analysis of base-case scenarios

**Figure 2:**
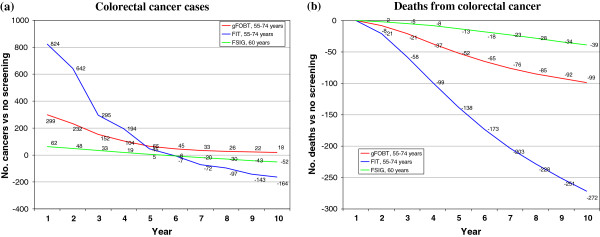
Relative analysis: estimated difference in numbers of (a) colorectal cancer cases diagnosed, and (b) deaths from colorectal cancer, in the population with screening versus a policy of no screening, for years 1–10, by screening scenario.

Compared to a policy of no screening, screening by gFOBT, FIT and FSIG all had potential to reduce deaths from colorectal cancer in the population within a decade of implementation. This reduction was evident from the second year, and was greatest for FIT-based screening (Figure 2). FIT-based screening at ages 55–74 would result in 272 colorectal cancer deaths avoided in the population in year 10 (26% fewer deaths than under no screening). In the first five years of screening, all three scenarios would generate an overall increase in colorectal cancer cases (Figure 2). After this time, with screening based on FIT or FSIG, the number of cases would fall below the number expected with no screening; this fall would be greatest with FIT (164 cases averted in year 10; 7% reduction compared to no screening).

## Discussion

### Value of resource modelling

The resource modelling described in this paper could be viewed as a particular type of budget impact analysis, with costs expressed in terms of resources and harms rather than monetary units. As in budget impact analyses [8], we focussed on the short-term health service impact of adopting technologies and generated results specific to the actual target population. Like budget impact analyses, the types of analyses shown here are intended to address the practical needs of both decision-makers and health service planners. They are also intended to complement more conventional cost-effectiveness analyses. In fact, these analyses did inform decision making in Ireland: a population-based colorectal cancer screening programme in 2013 commenced (see below) [51], and the estimates were used in planning the implementation of the programme. Our findings suggested that the annual endoscopy requirements associated with screening the 55–74 age-group by biennial FIT would be difficult, if not impossible, for the health services to meet in the short-term. However, the magnitude of the health gains that could be achieved within five years of screening implementation meant that FIT-based screening remained the preferred option of policy-makers, despite the lower resource requirements for gFOBT and FSIG. Therefore, the policy decision was to introduce biennial FIT screening; initially the programme will invite individuals aged 60–69 to be screened with the intention of eventually rolling-out screening to the full 55–74 age-range.

### Delivering sufficient capacity

The few previous analyses of the health service impact of colorectal cancer screening [12,52,53] all noted that the ability of the health services to deliver sufficient endoscopy services was a crucial determinant of programme feasibility. In England, for example, although screening based on once-only FSIG was the most cost-effective option [12], the associated requirements for endoscopy facilities and staff were considered impossible to meet in the short-term: instead, a programme based on gFOBT was implemented.

In this study, we found that a programme based on FIT the option considered most cost-effective [9] – would require substantially more resources for colonoscopy, CT colonography, and other diagnostic procedures, than one based on gFOBT (with FIT as the second-line test in those who test weakly positive) or FSIG. FIT-based screening will generate demand for 11,000-15,000 additional colonoscopies annually in Ireland. In Ireland, in 2008, 44,000 publically-funded colonoscopies were performed, 2,000 of these under the National Treatment Purchase Fund. This fund provides for procedures to be undertaken in private facilities in the event of long-waiting times in the public system. In April 2009 more than 2,300 individuals had been waiting for a colonoscopy for more than three months [54]. These figures suggest there is little spare capacity in the service, at least under current working patterns. They also justify the assumption, inherent in our analysis, that meeting the demand for “screening-generated” colonoscopies would require additional capacity over and above existing services.

If not prepared for, increases in colonoscopy demand can increase waiting times [3]. Studies of screening programmes for other cancers have found that the period between having a positive screening test and waiting for diagnostic follow-up can be a time of considerable anxiety for the individuals concerned (see, for example, [55]). Although this issue does not appear to have been investigated to any great extent for colorectal screening, it is entirely plausible that many people will be distressed or anxious in the time between receiving a positive colorectal screening test result and attending for colonoscopy. Since anxiety and distress can be considered a cost of screening, serious consideration should be given to ensuring that as many people as possible undergo diagnosis and treatment (if required) as quickly as possible. Thus, while recent studies continue to build the clinical case for screening with FIT in preference to other tests [56-59], the implication of our results is that any FIT-based programme – in Ireland or elsewhere – will have to plan carefully how to deliver sufficient services for initial diagnosis and ongoing surveillance. The types of analyses illustrated here, and elsewhere [60], could usefully inform that planning process.

Strategies that might be used to help address endoscopy capacity and other resource challenges include restricting screening to a narrower age-range, and/or gradually phasing in screening across the full age-range. Although restricting FIT-based screening to those aged 55–64 would reduce resource requirements by around 40%, our cost-effectiveness analyses concluded that, when fully implemented this strategy would be slightly less desirable than screening the 55–74 age group [9]. This suggests that gradual roll-out across the full age-range would be preferable, and indeed this is what has been implemented in Ireland, albeit in a different age-group initially to that considered here [51]. However, it is worth remembering that the short-term health gains achieved with such phased-in screening will be lower than if the full age-group was screened from the outset.

### Safety

Safety is a key consideration in the implementation of any screening programme [6]. The chances of bowel perforation and death are, in fact, the major reasons why some – including the NCSS in Ireland – consider colonoscopy unsuitable as a primary screening tool [61]. Colonoscopy is, however, the main option for diagnostic follow-up of those with positive screening tests. Our analysis suggest that the much higher numbers of colonoscopies generated by FIT-based screening than the other options will result in a higher number of adverse events. A higher adverse event rate was also evident in our cost-effectiveness analysis [9]. However, the advantage of the results presented here is that they express the actual numbers of events that might be expected to occur in the real-world eligible population: these values are likely to be more informative for the purposes of assessing and managing risk.

### Screening uptake

A screening test must be acceptable to the population [6] and one of the main measures of acceptability is screening uptake. As might be expected, the resource requirements and health gains attainable with screening were highly dependent on estimates of uptake. For all screening tests, if actual uptake was higher than our base-case estimates more cancers would be averted; however, resource requirements would be higher and there would be more colonoscopy-related adverse events. On the other hand, if uptake was lower than the base-case value, resource requirements would be lower and there would be fewer adverse events, but there would also be lower health gains.

Since it is impossible to be entirely certain what uptake might be attained for any screening test in any population, base-case values were chosen to be conservative. For the faecal tests, the base-case estimate of 53% was based on the second round of the UK pilot programmes [34,35]. Although this is lower than considered desirable by the NCSS in Ireland (60%) [62], it is higher than in some other European programmes and pilot programmes [58,63-65]. In Ireland, 51% of almost 10,000 Dublin residents invited to complete a FIT did so [66], suggesting the base-case estimate may be reasonably realistic. For FSIG, there was no local data to inform what level of uptake might be realistic. Although participation rates of 55% and higher have been achieved in some settings [40,67], generally uptake has been lower [58,63]. We decided to derive our base-case value from the large UK Flexible Sigmoidoscopy Trial [38] to provide some consistency with the source of the estimates of uptake for the faecal tests.

Since we conducted our analysis evidence has accrued that uptake may be higher with FIT than gFOBT [68], although this has not been a universal finding [59]. If FIT is more acceptable than gFOBT, our estimates of the resource requirements for FIT relative to gFOBT may be conservative. However, it is worth noting that the difference in numbers of screening-related harms may also be under-estimated.

### Limitations

Although parameter values were based on review of the best available evidence, as with all modelling studies, some uncertainty remains. Even for gFOBT, which has been shown to reduce mortality in several trials [69], performance characteristics remain uncertain. This is particularly true for newer versions of the test [70]. For FIT, the available tests have heterogeneous performance characteristics [70] and for FSIG, information on sensitivity and specificity is limited [18,20,33]. For all tests, assessment of true sensitivity and specificity is further complicated by the fact that the reference (gold) standard, colonoscopy, misses lesions [41]. We addressed this uncertainty through sensitivity analyses, and found that varying specificity, in particular, had a notable impact on requirements for diagnostic investigations. Hence, if the true test specificity differs from the values used here, the resource requirements and health outcomes might differ from those estimated.

We assumed, for gFOBT and FIT, that sensitivity and specificity remained constant across all screening rounds. Recent evidence suggests that the ability of gFOBT-based programmes to identify cancers might decline with repeated screenings [71]. The same may be true for FIT. If so, this would suggest that we may have over-estimated the health gains associated with screening programmes based on FIT and gFOBT, although the extent of this over-estimation is difficult to quantify from currently available evidence.

We evaluated a strategy that combined gFOBT with reflex FIT, instead of the more conventional approach of reflex gFOBT. This was because second-line FIT has been shown to limit the number of referrals to colonoscopy [72,73]. Finding effective strategies to “adjust” screening when colonoscopy capacity is limited is very topical [60]. Although gFOBT with reflex FIT is currently not widely used, our results indicate that it could be an attractive option for delivering population screening in settings where capacity for diagnostic investigation is expected to be limited.

We did not estimate resources required for adjuvant chemotherapy in screen-detected colon cancers, or for follow-up after resection (which can involve annual colonoscopy or CT colonography for five years) since responsibility for providing these would most likely fall outwith the screening programme/NCSS. Facilities would, however, need to be provided elsewhere in the health services, so this distinction is somewhat arbitrary.

## Conclusions

This study illustrates how resource modelling complements cost-effectiveness analyses and can help inform health service decision making and planning. Our results show that the introduction of FIT-based screening would quite quickly generate attractive health outcomes – in terms of reduced numbers of colorectal cancer cases and deaths – but that it has heavy resource requirements. These heavy requirements could impact on the feasibility of any programme based on this screening modality. Staggered age-based roll-out of FIT-based screening would allow time to increase endoscopy capacity to meet requirements. Overall out findings suggest that there are likely to be many challenges ahead in delivering a high-quality, safe, and acceptable screening programme to the population of Ireland.

## Abbreviations

CT: Computed tomography; FIT: Faecal immunochemical test; FSIG: Flexible sigmoidoscopy; gFOBT: guaiac-based Faecal Occult Blood; HSE: Health Service Executive; LYS: Life years saved; MRI: Magnetic resonance imaging; NCSS: National Cancer Screening Service; PET: Positron emission tomography; QALY: Quality adjusted life year; UK: United Kingdom; WHO: World Health Organisation.

## Competing interests

None of the authors have any conflicts of interest to declare.

## Authors’ contributions

All authors were involved in the design of the study, interpretation of data and commenting critically on the manuscript. In addition, LS co-ordinated the study, drafted the initial version of the manuscript, and undertook systematic reviews together with LT, AOC and CU. LT, CU and MB collated cost data. PT, JC and SW developed and modified the Markov model. CW provided mathematical and statistical input. All authors read and approved the final manuscript.

## Pre-publication history

The pre-publication history for this paper can be accessed here:

http://www.biomedcentral.com/1472-6963/13/105/prepub

## Supplementary Material

Additional file 1: Figure S1 Simplified diagram of Markov states in natural history mode. Click here for file

Additional file 2: Table S1 Sensitivity analysis: estimated numbers of diagnostic colonoscopies required and screen-detected cancers, for years one and 10, by screening scenario, as test sensitivity, test specificity and screening uptake vary around base-case values. Click here for file

Additional file 3: Table S2 Scenario analysis: estimated screening-related resource use and health outcomes by year for alternative FIT implementation options: biennial FIT at 55-64 years, biennial FIT at 55-74 years (medium roll-out), and biennial FIT at 55-74 years (slow roll-out)^1^. Click here for file

## References

[B1] Council of the European Union Council recommendation of 2 December 2003 on cancer screening (2003/878/EC) Off J Eur Union 2003 L327 34 38

[B2] U.S. Preventive Services Task Force Screening for colorectal cancer: U.S. Preventive Services Task Force recommendation statement Ann Intern Med 2008 149 627 637 18838716 10.7326/0003-4819-149-9-200811040-00243

[B3] Kavanos P Schurer W Dynamics of colorectal cancer management in 17 countries Eur J Health Econ 2010 10 Suppl 1 S115 S129 20012129 10.1007/s10198-009-0201-2

[B4] Whitlock EP Lin JS Liles S Beil TL Fu R Screening for colorectal cancer: a targeted, updated systematic review for the U.S. Preventive Services Task Force Ann Intern Med 2008 149 638 658 10.7326/0003-4819-149-9-200811040-00245 18838718

[B5] Landsdorp-Vogelaar I Knudsen AB Brenner H Cost-effectiveness of colorectal cancer screening- an overview Best Pract Res Clin Gastrenterol 2010 24 439 449 10.1016/j.bpg.2010.04.004 PMC2939039 20833348

[B6] Wilson JMG Junger G Principles and practice of screening for disease 1968 Geneva: WHO

[B7] Neuman P Budget impact analyses get some respect Value Health 2007 10 324 325 10.1111/j.1524-4733.2007.00237.x 17888096

[B8] Mauskopf JA Sullivan SD Annemans L Caro J Mukllins CD Nuijten M Orlewska E Watkins J Trueman P Principles of good practice of budget impact analysis: report of the ISPOR task force on good research practices – budget impact analysis Value Health 2007 10 336 347 10.1111/j.1524-4733.2007.00187.x 17888098

[B9] Sharp L Tilson L Whyte S O’Ceilleachair A Walsh C Usher C Tappenden P Chilcott J Staines A Barry M Comber H Cost-effectiveness of population-based screening for colorectal cancer: a comparison of guaiac-based faecal occult blood testing, faecal immunochemical testing and flexible sigmoidoscopy Br J Cancerin press 10.1038/bjc.2011.580 PMC3305953 22343624

[B10] Sharp L Walsh C Whyte S Tilson L O'Ceilleachair A Walsh C Usher C Tappenden P Chilcott J Staines A Barry M Comber H Letter to the editor Br J Cancer 2013 108 1211 1212 10.1038/bjc.2012.491 23392086 PMC3619055

[B11] Atkin WS Saunders BP Surveillance guidelines after removal of colorectal adenomatous polyps Gut 2002 51 Supplement 5 v6 v9 10.1136/gut.51.suppl_5.v6 12221031 PMC1867736

[B12] Tappeden P Chilcott J Eggington S Patrick J Sakai M Karnon J Option appraisal of population-based colorectal cancer screening programmes in England Gut 2007 56 677 684 10.1136/gut.2006.095109 17142648 PMC1942136

[B13] Allison JE Feldman R Tekawa IS Hemoccult screening in detecting colorectal neoplasm: sensitivity, specificity, and predictive value. Long-term follow-up in a large group practice setting Ann Intern Med 1990 112 328 33 10.7326/0003-4819-112-5-328 2407166

[B14] Castiglione G Grazzini G Poli A Bonardi R Ciatto S Hemoccult sensitivity estimate in a screening program for colorectal cancer in the Province of Florence Tumori 1991 77 243 5 1862554 10.1177/030089169107700312

[B15] Foley DP Dunne P Dervan PJ Callaghan TW Crowe J Lennon JR Left-sided colonoscopy and haemoccult screening for colorectal neoplasia Eur J Gastroenterol 1992 4 925 36

[B16] Allison JE Tekawa IS Ransom LJ Adrain AL A comparison of fecal occult-blood tests for colorectal-cancer screening N Engl J Med 1996 334 155 9 10.1056/NEJM199601183340304 8531970

[B17] Brevinge H Lindholm E Buntzen S Kewenter J Screening for colorectal neoplasia with faecal occult blood testing compared with flexible sigmoidoscopy directly in a 55–56 years’ old population Int J Colorect Dis 1997 12 291 5 10.1007/s003840050108 9401844

[B18] Lieberman DA Weiss DG Veterans Affairs Cooperative Study Group 380 One-time screening for colorectal cancer with combined fecal occult-blood testing and examination of the distal colon N Engl J Med 2001 345 555 560 10.1056/NEJMoa010328 11529208

[B19] Niv Y Lev-El M Fraser G Abuksis G Tamir A Protective effect of faecal occult blood test screening for colorectal cancer: worse prognosis for screening refusers Gut 2002 50 33 7 10.1136/gut.50.1.33 11772964 PMC1773062

[B20] Sung JJ Chan FK Leung WK Wu JC Lau JY Ching J To KF Lee YT Luk YW Kung NN Kwok SP Li MK Chung SC Screening for colorectal cancer in Chinese: comparison of fecal occult blood test, flexible sigmoidoscopy, and colonoscopy Gastroenterol 2003 124 608 14 10.1053/gast.2003.50090 12612899

[B21] Collins JF Lieberman DA Durbin TE Weiss DG Veterans Affairs Cooperative Study #380 Group Accuracy of screening for fecal occult blood on a single stool sample obtained by digital rectal examination: a comparison with recommended sampling practice Ann Intern Med 2005 142 81 5 10.7326/0003-4819-142-2-200501180-00006 15657155

[B22] Itoh M Takahashi K Nishida H Sakagami K Okubo T Estimation of the optimal cut off point in a new immunological faecal occult blood test in a corporate colorectal cancer screening programme J Med Screen 1996 3 66 71 8849762 10.1177/096914139600300204

[B23] Chen K Jiao DA Zheng S Zhou S Yu H Diagnostic value of fecal occult blood testing for screening colorectal cancer China Natl J New Gastroenterol 1997 3 166 8 10.3748/wjg.v3.i3.166 PMC4842878 27239137

[B24] Nakama H Fattah AS Zhang B Kamijo N Uehara Y Association of diverticulosis coli and vascular ectasias and the results of fecal occult blood test Hepatogastroenterol 2000 47 1277 1279 11100332

[B25] Nakama H Zhang B Fattah AA Kamijo N Zhang X Characteristics of colorectal cancer that produce positive immunochemical occult blood test results on stool obtained by digital rectal examination Can J Gastroenterol 2001 15 227 30 11331923 10.1155/2001/468125

[B26] Cheng TI Wong JM Hong CF Cheng SH Cheng TJ Shieh MJ Lin YM Tso CY Huang AT Colorectal cancer screening in asymptomaic adults: comparison of colonoscopy, sigmoidoscopy and fecal occult blood tests J Formos Med Assoc 2002 101 685 90 12517041

[B27] Gondal G Grotmol T Hofstad B Bretthauer M Eide TJ Hoff G The Norwegian colorectal cancer prevention (NORCCAP) screening study: baseline findings and implementations for clinical work-up in age groups 50–64 years Scand J Gastroenterol 2003 38 635 42 10.1080/00365520310003002 12825872

[B28] Liu HH Huang TW Chen HL Wang TH Lin JT Clinicopathologic significance of immunohistochemical fecal occult blood test in subjects receiving bidirectional endoscopy Hepatogastroenterol 2003 50 1390 1392 14571744

[B29] Morikawa T Kato J Yamaji Y Wada R Mitsushima T Shiratori Y A comparison of the immunochemical fecal occult blood test and total colonoscopy in asymptomatic population Gastroenterol 2005 129 422 8 10.1016/j.gastro.2005.05.056 16083699

[B30] Nakazato M Yamano H Matsushita H Sato K Fujita K Yamanaka Y Imai Y Immunologic fecal occult blood test for colorectal cancer screening Jap Med Assoc J 2006 49 203 7

[B31] Allison JE Sakoda LC Levin TR Tucker JP Tekawa IS Cuff T Pauly MP Shlager L Palitz AM Zhao WK Schwartz JS Ransohoff DF Selby JV Screening for colorectal neoplasms with new fecal occult blood tests: update on performance characteristics J Natl Cancer Inst 2007 99 1462 70 10.1093/jnci/djm150 17895475

[B32] Morikawa T Kato J Yamaji Y Wada R Mitsushima T Sakaguchi K Shiratori Y Sensitivity of immunochemical fecal occult blood test to small colorectal adenomas Am J Gastroenterol 2007 102 2259 64 10.1111/j.1572-0241.2007.01404.x 17617203

[B33] Rozen P Ron E Fireman Z Hallak A Grossman A Baratz M Rattan J Gilat T The relative value of fecal occult blood tests and flexible sigmoidoscopy in screening for large bowel neoplasia Cancer 1987 60 2553 8 10.1002/1097-0142(19871115)60:10<2553::AID-CNCR2820601034>3.0.CO;2-S 3664435

[B34] Weller D Moss S Butler P Campbell C Coleman D Melia J Robertson R English pilot of bowel cancer screening: an evaluation of the second round, Final report to the Department of Health 2006 Edinburgh: University of Edinburgh

[B35] Information Services Division Summary of the key performance indicators (KPIs) used to monitor and evaluate the Scottish bowel screening pilot 2008 Edinburgh: NHS National Services Scotland

[B36] Malila N Oivanen T Hakama M Implementation of colorectal cancer screening in Finland: experiences from the first three years of a public health programme Z Gastroenterol 2007 45 1 4 10.1055/s-2007-963490 18368636

[B37] Segnan N Senore C Andreoni B Azzoni A Bisanti L Cardelli A Castiglione G Crosta C Ederle A Fantin A Ferrari A Fracchia M Ferrero F Gasperoni S Recchia S Risio M Rubeca T Saracco G Zappa M SCORE3 Working Group-Italy Comparing attendance and detection rate of colonoscopy with sigmoidoscopy and FIT for colorectal cancer screening Gastroenterol 2007 132 2304 2012 10.1053/j.gastro.2007.03.030 17570205

[B38] UK Flexible Sigmoidoscopy Screening Trial Investigators Single flexible sigmoidoscopy screening to prevent colorectal cancer: baseline findings of a UK multicentre randomised trial Lancet 2002 359 1291 300 11965274 10.1016/S0140-6736(02)08268-5

[B39] Gray M Pennington CR Screening sigmoidoscopy: a randomised trial of invitation style Health Bull (Edinb) 2000 58 137 140 12813842

[B40] Bretthauer M Gondal G Larsen IK Carlsen E Eide TJ Grotmol T Skovlund E Tveit KM Vatn MH Hoff G Design, organization and management of a controlled population screening study for detection of colorectal neoplasia: attendance rates in the NORCCAP study (Norwegian Colorectal Cancer Prevention) Scand J Gastroenterol 2002 37 568 573 10.1080/00365520252903125 12059059

[B41] van Rijn JC Reitsma JB Stoker J Bossuyt PM van Deventer SJ Dekker E Polyp miss rate determined by tandem colonoscopy: a systematic review Am J Gastroenterol 2006 101 343 50 10.1111/j.1572-0241.2006.00390.x 16454841

[B42] Bressler B Paszat LF Chen Z Rothwell DM Vinden C Rabeneck L Rates of new or missed colorectal cancers after colonoscopy and their risk factors: a population-based analysis Gastroenterol 2007 132 96 102 10.1053/j.gastro.2006.10.027 17241863

[B43] Cotton PB Durkalski VL Pineau BC Palesch YY Mauldin PD Hoffman B Vining DJ Small WC Affronti J Rex D Kopecky KK Ackerman S Burdick JS Brewington C Turner MA Zfass A Wright AR Iyer RB Lynch P Sivak MV Butler H Computed tomographic colonography (virtual colonoscopy): a multicenter comparison with standard colonoscopy for detection of colorectal neoplasia J Am Med Assoc 2004 291 1713 9 10.1001/jama.291.14.1713 15082698

[B44] Halligan S Altman DG Taylor SA Mallett S Deeks JJ Bartram CI Atkin W CT colonography in the detection of colorectal polyps and cancer: systematic review, meta-analysis, and proposed minimum data set for study level reporting Radiology 2005 237 893 904 10.1148/radiol.2373050176 16304111

[B45] Mulhall BP Veerappan GR Jackson JL Meta-analysis: computed tomographic colonography Ann Intern Med 2005 142 635 50 10.7326/0003-4819-142-8-200504190-00013 15838071

[B46] Johnson CD Chen MH Toledano AY Heiken JP Dachman A Kuo MD Menias CO Siewert B Cheema JI Obregon RG Fidler JL Zimmerman P Horton KM Coakley K Iyer RB Hara AK Halvorsen RA Jr Casola G Yee J Herman BA Burgart LJ Limburg PJ Accuracy of CT colonography for detection of large adenomas and cancers N Engl J Med 2008 359 1207 1217 10.1056/NEJMoa0800996 18799557 PMC2654614

[B47] Winawer SJ Flehinger BJ Schottenfeld D Miller DG Screening for colorectal cancer with fecal occul blood testing and sigmoidoscopy J Natl Cancer Inst 1993 85 1311 8 10.1093/jnci/85.16.1311 8340943

[B48] Gatto NM Frucht H Sundararajan V Jacobson JS Grann VR Neugut AI Risk of perforation after colonoscopy and sigmoidoscopy: a population-based study J Natl Cancer Inst 2003 95 230 6 10.1093/jnci/95.3.230 12569145

[B49] Dafnis G Ekbom A Pahlman L Blomqvist P Complications of diagnostic and therapeutic colonoscopy within a defined population in Sweden Gastrointest Endosc 2001 54 302 9 10.1067/mge.2001.117545 11522969

[B50] Whyte S Walsh C Chilcott J Bayesian calibration of a natural history model with application to a population model for colorectal cancer Med Decis Making [Epub ahead of print] 10.1177/0272989X10384738 21127321

[B51] National Cancer Screening Service Update on Progress on the Implementation of the National Colorectal Cancer Screening Programme 2011 Dublin: NCSS Available at http://www.cancerscreening.ie/bowel-screening.html [Accessed 21st March 2013]

[B52] O’Leary BA Olynyk JK Neville AM Platell CF Cost-effectiveness of colorectal cancer screening: comparison of community-based flexible sigmoidoscopy with fecal occult blood testing and colonoscopy J Gastroenterol Hepatol 2004 19 38 47 10.1111/j.1440-1746.2004.03177.x 14675241

[B53] Ho C Heitman S Membe SK Morrison A Moulton K Manns B Au F Reed M Hilsden R Computed tomographic colonography for colorectal cancer screening in an average risk population: systematic review and economic evaluation 2008 Ottawa: Canadian Agency for Drugs and Technologies in Health

[B54] Health Information and Quality Authority Report of the evaluation of use of resources in the national population-based cancer screening programmes and associated services 2009 Dublin: HIQA Available at URL http://www.hiqa.ie/system/files/Colorectal_cancer_evaluation_report_HTA.pdf [Accessed 21st March 2013]

[B55] Gray NM Sharp L Cotton SC Masson LF Little J Walker LG Avis M Philips Z Russell I Whynes D Cruickshank M Woolley CM TOMBOLA group Psychological effects of a low-grade abnormal cervical smear test result: anxiety and associated factors Br J Cancer 2006 94 1253 62 10.1038/sj.bjc.6603086 16622462 PMC2361408

[B56] Guittet L Bouvier V Mariotte N Vallee JP Arsène D Boutreux S Tichet J Launoy G Comparison of screening for colorectal cancer in a general average immunochemical faecal occult blood test in a general average risk population Gut 2007 56 210 4 10.1136/gut.2006.101428 16891354 PMC1856766

[B57] Van Rossum LG van Rijn AF Verbeek AL van Oijen MG Laheij RJ Fockens P Jansen JB Adang EM Dekker E Colorectal cancer screening comparing no screening, immunochemical and guaiac fecal occult blood tests: a cost-effectiveness analysis Int J Cancer 2011 128 1908 17 10.1002/ijc.25530 20589677

[B58] Hol L van Leerdam ME van Ballegooijen M van Vuuren AJ van Dekken H Reijerink JC van der Togt AC Habbema JD Kuipers EJ Screening for colorectal cancer: randomized trial comparing guaiac-based and immunochemical faecal occult blood testing and flexible sigmoidoscopy Gut 2010 59 62 68 10.1136/gut.2009.177089 19671542

[B59] Levi Z Birkenfeld S Vilkin A Bar-Chana M Lifshitz I Chared M Maoz E Niv Y A higher detection rate for colorectal cancer and advanced adenomatous polyp for screening with immunochemical fecal occult blood test than guaiac fecal occult blood test, despite lower compliance rate. A prospective, controlled, feasibility study Int J Cancer 2011 128 2415 24 10.1002/ijc.25574 20658527

[B60] Wilschut JA Habbema DF van Leerdam ME Hol L Lansdorp-Vogelaar I Kuipers EJ Ballegooijen M Fecal occult blood testing when colonoscopy capacity is limited J Natl Cancer Inst 2011 103 1741 51 10.1093/jnci/djr385 22076285

[B61] Ransohoff DF Screening colonoscopy in balance. Issues of implementation Gastroenterol Clin North Am 2002 31 1031 44i 10.1016/S0889-8553(02)00059-6 12489276

[B62] National Cancer Screening Service First report of the National Cancer Screening Service Expert Advisory Group on colorectal cancer screening 2007 Dublin: National Cancer Screening Service

[B63] Zorzi M Baracco S Fedato C Grazzini G Naldoni C Sassoli De Biachi P Senore C Visioli CB Cogo C Screening for colorectal cancer in Italy: 2008 survey Epidemiol Prev 2010 34 5–6 Suppl 4 53 72 21220837

[B64] Peris M Espinas JA Munoz L Navarro M Binefa G Borras JM Catalan Colorectal Cancer Screening Pilot Programme Group Lesson learnt from a population-based pilot programme for colorectal cancer screening in Catalonia (Spain) J Med Screen 2007 14 81 86 10.1258/096914107781261936 17626707

[B65] Kis RK National colorectal cancer screening program in the Republic of Croatia – experiences, outcomes and obstacles in the program implementation in the Medimurje County Acta Med Croatica 2010 64 363 74 21692260

[B66] McNemara D Qasim A Lee N Condon C O’Morain C Round one of the adelaide and meath hospital/trinity college colorectal cancer screening programme: programme report and analysis based on established international key performance indices Ir J Med Sci 2011 180 549 52 10.1007/s11845-010-0650-8 21264524

[B67] Brotherstone H Vance M Edwards R Miles A Robb KA Evans RE Wardle J Atkin W Uptake of population-based flexible sigmoidoscopy screening for colorectal cancer: a nurse-led feasibility study J Med Screen 2007 14 2 76 80 10.1258/096914107781261972 17626706 PMC2817449

[B68] Vart G Banzi R Minozzi S Comparing participation rates between immunochemical and guaiac faecal occult blood tests: a systematic review and meta-analysis Prev Med 2012 55 87 92 10.1016/j.ypmed.2012.05.006 22634386

[B69] Hewitson P Glasziou P Irwig L Towler B Watson E Screening for colorectal cancer using the faecal occult blood test Hemoccult Cochrane Database Syst Rev 2007 24 CD001216 10.1002/14651858.CD001216.pub2 PMC6769059 17253456

[B70] Soares-Weiser K Burch J Duffy S St John J Smith S Westwood M Kleijnen J Diagnostic accuracy and cost-effectiveness of faecal occult blood tests used in screening for colorectal cancer: a systematic review 2007 York: Centre for Reviews and Dissemination 10.1258/096914107782066220 17925085

[B71] Steele RJC McClements P Watling C Libby G Weller D Brewster D Black R Carey FA Fraser CG Interval cancers in a FOBT-based colorectal cancer population screening programme: implications for stage, gender and tumour site Gut 2012 61 576 61 10.1136/gutjnl-2011-300535 21930729

[B72] Fraser CG Matthew CM Mowat NA Wilson JA Carey FA Steele RJ Immunochemical testing of individuals positive for guaiac faecal occult blood test in a screening programme for colorectal cancer: an observational study Lancet Oncol 2006 7 127 31 10.1016/S1470-2045(05)70473-3 16455476

[B73] Fraser CG Mathew CM Mowat NA Wilson JA Carey FA Steele RJ Evaluation of a card collection-based faecal immunochemical test in screening for colorectal cancer using a two-tier reflex approach Gut 2007 56 1415 8 10.1136/gut.2007.119651 17309886 PMC2000260

