# Supplementary material for: Using resource modelling to inform decision making and service planning: the case of colorectal cancer screening in Ireland
Source: BMC Health Serv Res. 2013 Mar 19;13:105. doi: 10.1186/1472-6963-13-105 (PMC3637462; doi:10.1186/1472-6963-13-105)
Supplement: Additional file 1: Figure S1 — Simplified diagram of Markov states in natural history mode. [file 1472-6963-13-105-S1.doc]

Stage IV CRC

NORMAL

Low-risk adenoma(s)

Intermediate/high-risk adenoma(s)

Stage I CRC

Stage II CRC

Stage III CRC

DEATH

DEATH

low-risk polyp(s): <10 mm; intermediate/high-risk adenomas: ≥10mm; CRC=colorectal cancer
