# Supplementary material for: Using resource modelling to inform decision making and service planning: the case of colorectal cancer screening in Ireland
Source: BMC Health Serv Res. 2013 Mar 19;13:105. doi: 10.1186/1472-6963-13-105 (PMC3637462; doi:10.1186/1472-6963-13-105)
Supplement: Additional file 2: Table S1 — Sensitivity analysis: estimated numbers of diagnostic colonoscopies required and screen-detected cancers, for years one and 10, by screening scenario, as test sensitivity, test specificity and screening uptake vary around base-case values. [file 1472-6963-13-105-S2.doc]

**Supplementary table 1. Sensitivity analysis: estimated numbers of diagnostic colonoscopies required and screen-detected cancers, for years one and 10, by screening scenario, as test sensitivity, test specificity and screening uptake vary around base-case values**

| ***Year of programme*** |  |  |  | ***Year 1*** |  |  |  | ***Year 10*** |  |
| --- | --- | --- | --- | --- | --- | --- | --- | --- | --- |
| ***Screening scenario, parameter varied and resource estimated*** |  |  | ***gFOBT*** | ***FIT*** | ***FSIG*** |  | ***gFOBT*** | ***FIT*** | ***FSIG*** |
| **Test sensitivity^1^:** *No. of diagnostic colonoscopies required* |  |  |  |  |  |  |  |  |  |
|  | Low |  | 831 | 10,752 | 378 |  | 955 | 12,109 | 419 |
|  | Base-case |  | 967 | 11,095 | 381 |  | 1,103 | 12,414 | 423 |
|  | High |  | 1,102 | 11,290 | 385 |  | 1,243 | 12,577 | 427 |
| **Test sensitivity^1^:** *No. of screen-detected cancers* |  |  |  |  |  |  |  |  |  |
|  | Low |  | 252 | 805 | 60 |  | 279 | 668 | 74 |
|  | Base-case |  | 309 | 853 | 64 |  | 336 | 687 | 78 |
|  | High |  | 381 | 901 | 67 |  | 405 | 709 | 82 |
|  |  |  |  |  |  |  |  |  |  |
| **Test specificity^2^:** *No. of diagnostic colonoscopies required* |  |  |  |  |  |  |  |  |  |
|  | Low |  | 867 | 9,665 | - |  | 985 | 10,718 | - |
|  | Base-case |  | 967 | 11,095 | 381 |  | 1,103 | 12,414 | 423 |
|  | High |  | 1,096 | 12,524 | - |  | 1,254 | 14,108 | - |
|  |  |  |  |  |  |  |  |  |  |
| **Screening uptake^3^:** *No. of diagnostic colonoscopies required* |  |  |  |  |  |  |  |  |  |
|  | Low |  | 584 | 6,699 | 175 |  | 676 | 7,640 | 197 |
|  | Base-case |  | 967 | 11,095 | 381 |  | 1,103 | 12,414 | 423 |
|  | High |  | 1,278 | 14,653 | 489 |  | 1,439 | 16,165 | 551 |
| **Screening uptake^3^:** *No. of screen-detected cancers* |  |  |  |  |  |  |  |  |  |
|  | Low |  | 187 | 515 | 28 |  | 212 | 491 | 35 |
|  | Base-case |  | 309 | 853 | 64 |  | 336 | 687 | 78 |
|  | High |  | 409 | 1,126 | 78 |  | 430 | 790 | 98 |

^1^ Sensitivity values for adenomas and cancers varied simultaneously. For FOBT, for adenomas, low=10%, base-case=11%, high=12%; for FOBT, for cancers, low =31%, base-case=36%, high=42%; for FIT, for adenomas, low=19%, base-case=21%, high=22%; for FIT, for cancers, low=67%, base-case=71%, high=75%; for FSIG, for low-risk distal adenomas, low=60%, base-case=65%, high=70%; for FSIG, for intermediate/high-risk distal adenomas, low=68%, base-case=74%, high=78%; for FSIG, for distal cancers, low=85%, base-case=90%, high=95%

^2^ For FOBT, low=96%, base-case=97%, high=98%; for FIT, low=94%, base-case=95%, high=96%; for FSIG, low=90%, base-case=92%, high=95%; varying specificity does not affect number of cancers detected

^3^ For gFOBT and FIT, low=32%, base-case=53%, high=70%; for FSIG, low=24%, base-case=39% and high=67%

^4^ Sensitivity analysis not done for FSIG since referral for diagnostic colonoscopy only takes place for individuals with a “positive” FSIG examination (i.e. adenoma or cancer detected but not removed at FSIG).
