# Supplementary material for: Using resource modelling to inform decision making and service planning: the case of colorectal cancer screening in Ireland
Source: BMC Health Serv Res. 2013 Mar 19;13:105. doi: 10.1186/1472-6963-13-105 (PMC3637462; doi:10.1186/1472-6963-13-105)
Supplement: Additional file 3: Table S2 — Scenario analysis: estimated screening-related resource use and health outcomes by year for alternative FIT implementation options: biennial FIT at 55-64 years, biennial FIT at 55-74 years (medium roll-out), and biennial FIT at 55-74 years (slow roll-out)1. [file 1472-6963-13-105-S3.doc]

**Supplementary table 2. Scenario analysis: estimated screening-related resource use and health outcomes by year for alternative FIT implementation options: biennial FIT at 55-64 years, biennial FIT at 55-74 years (medium roll-out), and biennial FIT at 55-74 years (slow roll-out)^1^**

| **Year of programme** |  |  |  | **Year 1** |  |  |  | **Year 5** |  |  |  | **Year 10** |  |
| --- | --- | --- | --- | --- | --- | --- | --- | --- | --- | --- | --- | --- | --- |
| ***Screening scenario &***  ***outcome*** |  |  | ***FIT 55-64 years*** | ***Medium roll-out*** | ***Slow roll-out*** |  | ***FIT 55-64 years*** | ***Medium roll-out*** | ***Slow roll-out*** |  | ***FIT 55-64 years*** | ***Medium roll-out*** | ***Slow roll-out*** |
| **Screening-related resource use** |  |  |  |  |  |  |  |  |  |  |  |  |  |
| ***Screening tests*** | No. of kits sent out |  | 219,171 | 82,602 | 49,072 |  | 232,301 | 249,474 | 147,119 |  | 247,425 | 418,242 | 247,425 |
|  | No. of kits processed |  | 116,161 | 43,779 | 26,008 |  | 123,052 | 132,157 | 77,946 |  | 131,052 | 221,473 | 131,052 |
|  |  |  |  |  |  |  |  |  |  |  |  |  |  |
| ***COL/CTC*** | No. of diagnostic COL |  | 6,437 | 2,441 | 1,369 |  | 6,662 | 7,415 | 4,145 |  | 7,070 | 12,567 | 7,070 |
|  | No. of diagnostic CTC |  | 837 | 317 | 178 |  | 866 | 964 | 539 |  | 919 | 1,634 | 919 |
|  | No. of surveillance COL |  | 0 | 0 | 0 |  | 621 | 313 | 127 |  | 1,236 | 1,503 | 657 |
|  | No. of surveillance CTC |  | 0 | 0 | 0 |  | 81 | 41 | 16 |  | 161 | 195 | 85 |
|  |  |  |  |  |  |  |  |  |  |  |  |  |  |
| ***Pathology*** | No. of CRC and adenomas requiring pathology^2^ |  | 3,463 | 1,345 | 593 |  | 3,720 | 4,304 | 1,969 |  | 4,384 | 8,476 | 3,902 |
|  |  |  |  |  |  |  |  |  |  |  |  |  |  |
| ***CRC work-up***  ***and treatment*** | No. receiving PET scan |  | 38 | 15 | 6 |  | 31 | 43 | 17 |  | 33 | 75 | 32 |
|  | No. receiving MRI scan |  | [136](mailto:%3DProp_RC*@sum(D38:D41)) | 54 | 21 |  | [111](mailto:%3DProp_RC*@sum(D38:D41)) | 156 | 63 |  | [117](mailto:%3DProp_RC*@sum(D38:D41)) | 271 | 114 |
|  | No. receiving CT scan(s) |  | 377 | 150 | 59 |  | 309 | 434 | 175 |  | 325 | 754 | 317 |
|  | No. receiving TUS |  | 19 | 8 | 3 |  | 16 | 22 | 9 |  | 16 | 38 | 16 |
|  | No. receiving pre-operative radiotherapy^3^ |  | 86 | 34 | 13 |  | 66 | 96 | 38 |  | 69 | 164 | 68 |
|  | No. undergoing colorectal resection |  | 345 | 137 | 54 |  | 185 | 399 | 161 |  | 300 | 695 | 292 |
|  |  |  |  |  |  |  |  |  |  |  |  |  |  |
| **Screening-related health outcomes** |  |  |  |  |  |  |  |  |  |  |  |  |  |
| ***Harms^4^*** | No. with major bleeding following endoscopy |  | 28 | 10 | 6 |  | 30 | 32 | 18 |  | 35 | 59 | 32 |
|  | No. with perforation following endoscopy |  | 13 | 5 | 3 |  | 14 | 15 | 8 |  | 16 | 26 | 15 |
|  | No. of deaths from perforation following endoscopy |  | 1 | 0 | 0 |  | 1 | 1 | 0 |  | 1 | 1 | 1 |
|  |  |  |  |  |  |  |  |  |  |  |  |  |  |
| ***Screen-detected***  ***Ad & CRC*** | No. with adenoma(s)^5^ |  | 1,624 | 629 | 281 |  | 1,795 | 2,085 | 944 |  | 2,136 | 4,064 | 1,887 |
|  | No. with CRC^6^ |  | 377 | 150 | 59 |  | 309 | 434 | 175 |  | 325 | 754 | 317 |

Ad=Adenomas; COL=colonoscopy; CRC=colorectal cancer; CTC=CT colonography; gFOBT=guaiac-based faecal occult blood test; FIT= faecal immunochemical test; FSIG= flexible sigmoidoscopy; TUS= ultrasound; intermediate/high-risk=adenoma(s) ≥10mm; low-risk=adenoma(s) <10mm;

^1^ FIT ages 55-64, with full roll-out in year 1; medium implementation - 55 and 65 years invited in year 1, 55,57,65 and 67 in year2, 55,57,59,65,67,69, in year 3, etc; slow implementation- 55 years invited in year 1, 55 and 57 in year 2, 55, 57, 59 in year 3, etc

^2^ assuming average of 1.9 adenomas per person; includes screen-detected and surveillance-detected adenomas

^3^ includes radiotherapy given with or without chemotherapy

^4^ includes complications from diagnostic and surveillance endoscopy, including FSIG where relevant

^5^ includes individuals with screen-detected and surveillance-detected adenomas

^6^ includes individuals with CRC detected at screening and at surveillance
